# Control and value appraisals and online multiple‐text comprehension in primary school: The mediating role of boredom and the moderating role of word‐reading fluency

**DOI:** 10.1111/bjep.12448

**Published:** 2021-07-26

**Authors:** Daniela Raccanello, Elena Florit, Margherita Brondino, Antonio Rodà, Lucia Mason

**Affiliations:** ^1^ Department of Human Sciences University of Verona Italy; ^2^ Department of Information Engineering University of Padova Italy; ^3^ Department of Developmental Psychology and Socialization University of Padova Italy

**Keywords:** boredom, control, online multiple‐text comprehension, task‐value, word‐reading fluency

## Abstract

**Background:**

Online multiple‐text comprehension is a key skill of the 21st Century, yet the study of its relations with boredom in young students has been disregarded. Boredom is an achievement emotion expected to be predicted negatively by antecedents like control and value appraisals and to be associated to a negative performance. Notwithstanding its documented domain‐specificity, scarce attention has been paid to investigating these relations with primary‐school students in the reading domain, and specifically for online multiple‐text comprehension, and to how such relations are moderated by basic cognitive abilities.

**Aims:**

Considering separately two settings (homework, test), we studied the mediation of boredom in the relation between control‐value appraisals and online multiple‐text comprehension in primary‐school students, focusing on the moderating role of word‐reading fluency.

**Sample:**

Participants were 334 fourth and fifth graders.

**Methods:**

We evaluated students’ reading‐related self‐efficacy and task‐value, reading‐related boredom for homework and tests, word‐reading fluency, and online multiple‐text comprehension.

**Results:**

Path analyses revealed negative relations between control‐value appraisals and boredom for homework and tests, and between boredom and online multiple‐text comprehension for tests only. For the latter, word‐reading fluency moderated the relation between appraisals, boredom, and comprehension: Boredom negatively related to comprehension only for students with high word‐reading fluency.

**Conclusions:**

Findings are discussed focusing on antecedents of online multiple‐text comprehension as a literacy skill critical in the 21st Century. We underlined their implications for learning in general and specifically for the current educational changes due to the COVID‐19 pandemic.

## Background

In the last decade, the relevance of boredom for students’ well‐being and learning has been increasingly highlighted (Camacho‐Morles et al., 2021; Krannich et al., 2019; Pekrun, Lichtenfeld, Marsh, Murayama, & Goetz, 2017; Schwartze et al., 2020, 2021; Tze, Daniels, & Klassen, 2016). In line with Pekrun’s control‐value theory (CVT; Pekrun, 2006), recent research has documented the impact of boredom also on primary students’ school tasks about mathematics in most of the studies, and other domains like language or reading as well (for meta‐analyses on boredom and correlates, see Camacho‐Morles et al., 2021; Loderer, Pekrun, & Lester, 2020; Tze et al., 2016). However, the studies have neglected the relations of boredom with online multiple‐text comprehension, a key skill in the 21st Century in which technology‐based learning environments play a critical role for education (Lajoie, Pekrun, Azevedo, & Leighton, 2020). Currently, this ability has assumed even a greater relevance, considering the abrupt partial or full closure of schools and the shift to remote learning to which most of the students were forced worldwide due to the COVID‐19 pandemic (UNESCO, 2021). This makes it pivotal to know the reasons why boredom arises and of the consequences that it has on abilities essential for learning in technology‐based environments, for example understanding information from multiple sources or navigating the Internet. Such knowledge is essential to develop evidence‐based strategies aimed to mitigate boredom within education contexts.

Therefore, in this paper, we studied the mediating role of reading‐related boredom in the relation between reading‐related control and value appraisals and online multiple‐text comprehension in primary‐school students. While some researchers have investigated the role of primary students’ boredom in domains such as mathematics and more rarely for language or reading, only recently attention has been paid to studying appraisals or outcomes of boredom in technology‐based environments (Loderer et al., 2020). Anyway, most of such studies involved secondary‐school students, for example considering computer‐based collaborative problem‐solving, engagement in ‘gaming the system’ behaviours (i.e., using the affordances of a system to complete a task), learning math with multimedia software or intelligent tutoring systems (Baker et al., 2008; Camacho‐Morles, Slemp, Oades, Pekrun, & Morrish, 2019; Rodrigo et al., 2012; Sabourin & Lester, 2014; Tulis & Ainley, 2011). In contrast, primary‐school students were only rarely involved (intelligent tutoring systems; Robertson, Cross, Macleod, & Wiemer‐Hastings, 2004). Furthermore, to the best of our knowledge no studies have examined the contribution of a basic cognitive skill for reading comprehension, that is word‐reading fluency, in the interplay of boredom, its proximal appraisals, and online multiple‐text comprehension.

Therefore, we also explored the moderating role of word‐reading fluency for the relations between control and value appraisals, boredom, and online multiple‐text comprehension. In light of Pekrun’s assumptions on the domain‐specificity of achievement emotions (Pekrun, 2006), we examined two school settings, that is, homework and tests, in which boredom can be experienced in relation to reading activity.

### Online multiple‐text comprehension

From the last years of primary school onwards, students are increasingly required to construct meaning from multiple texts for various purposes. Among them, it is frequent comparing and reasoning about the information used to support a claim, and to express them in written tasks such as short essays, syntheses, and presentations (Bråten, Ferguson, Anmarkrud, & Strømsø, 2013; see Barzilai, Zohar, & Mor‐Hagani, 2018, for a review). In today’s information‐rich world, in fact, students frequently acquire knowledge by comprehending the content of different texts, written by different sources (e.g., authors of the texts) and often reporting conflicting information on the same topic (Rouet, Britt, & Durik, 2017).

A number of theoretical models have been proposed to explain comprehension of multiple texts. The integrated framework of multiple texts (IF‐MT; List & Alexander, 2019) is a recent account that integrates and extends early models on multiple‐text comprehension. According to the IF‐MT , multiple‐text comprehension results from three stages: preparation, execution, and production. The latter stage results in external products which accomplish the requirements of a specific task (e.g., to write an argumentative essay). Such products are the outcomes of the execution stage that comprises three levels of internal representation (Rouet et al., 2017). The first level is the situation model (Kintsch, 1988) that allows the representation and integration of information within each text. The second level is the intertext model that involves the representation of text sources and their relations with text content. The third level is the integrated mental model that allows the integration of text content from multiple texts into a coherent representation, which often requires reconciling discrepancies. Both the production and the execution stages are influenced by the preparatory or ‘default stance’ (i.e., an orientation) towards multiple‐text comprehension tasks adopted during the preparation stage. This stance is affected by objective context requirements (e.g., the task and resources, or the obstacles in the environment) and individual factors (e.g., cognitive and affective factors) influencing task perception.

Argumentative short essays are considered as hybrid reading‐writing tasks (Barzilai et al., 2018). They have been used by previous studies in upper primary‐ and lower secondary‐school students and shown to be suitable to sustain deep level understanding of multiple texts (Florit, Cain, & Mason, 2019; Kingsley, Cassady, & Tancock, 2015; LaRusso et al., 2016). Usually, hybrid tasks are untimed tasks that are assessed by considering measures of global coherence, which are included in writing quality measures. These measures have been shown to involve a number of cognitive factors that are common to single and multiple‐text comprehension (Arfé, Dockrell, & De Bernardi, 2016; Kim & Schatschneider, 2017).

Theoretical models of multiple‐text comprehension have been applied to both printed (presented on paper) and online (presented on screen) texts. The role played by reading medium has been addressed only recently and mainly considering single texts. In particular, three recent meta‐analyses have shown that reading expository or informational texts on paper leads to greater text comprehension than reading them on screen (Clinton, 2019; Delgado, Vargas, Ackerman, & Salmerón, 2018; Kong, Seo, & Zhai, 2018). This result has been accounted for by the shallow hypothesis (Annisette & Lafreniere, 2017). According to this hypothesis, today’s students are more inclined towards superficial processing when reading digitally because of the increasing interaction with technology that is characterized by speed and multitasking. Shallow processing is at the basis of decreased deep comprehension in digital environments (Clinton, 2019; Delgado et al., 2018). Of note is that this issue regards studies that mainly involved university or secondary school students and, although to a lesser extent, also students at the end of primary education.

To date, the few studies on multiple‐text comprehension in primary‐school students have mainly considered printed texts and investigated the role of cognitive factors, both basic such as word‐reading fluency, and more complex such as prior knowledge, comprehension monitoring, theory of mind, and single‐text comprehension (Davis, Huang, & Yi, 2017; Florit et al., 2019; Florit, De Carli, Giunti, & Mason, 2020; LaRusso et al., 2016). The role of contextual factors, such as the presence of conflicts in the reading materials and prompts for taking into account the source of information, have also been investigated (Paul, Stadtler, & Bromme, 2019; Potocki et al., 2020). In contrast, the contribution of affective factors, especially emotions, to multiple‐text comprehension in young readers of primary school has been disregarded. Empirical investigations have explored the role of affect in terms of motivation (i.e., beliefs about control or value, or interest), mood, epistemic emotions, or physiological reactions only in older students (Bråten et al., 2013; Bråten, Anmarkrud, Brandmo, & Strømsø, 2014; Muis, Pekrun, et al., 2015; Scrimin & Mason, 2015; Mason, Scrimin, Zaccoletti, Tornatora & Goetz, 2018; Trevors, Muis, Pekrun, Sinatra, & Muijselaar, 2017). Nevertheless, knowledge is still lacking on how boredom influences multiple‐text comprehension, and specifically online multiple‐text comprehension in primary‐school students.

### The control‐value theory of achievement emotions: The case of boredom

#### Boredom and reading

The emotion of boredom can play a relevant role in reading in primary‐school students; yet this emotion is partially neglected in the extant literature (as an exception, for comprehension of single texts in lower secondary‐school students, see Zaccoletti, Altoè, & Mason, 2020). We may legitimately expect that in reading activity boredom may regard at least three different aspects. These are related to the specific skills required to be good readers and comprehenders, to the content of the reading materials, and also to the reading medium itself, that is, traditional or technological. We also know that in the domain of native language, which includes the sub‐domain of reading, primary students’ boredom is more frequent as age increases (Raccanello, Brondino, & De Bernardi, 2013; Raccanello, Brondino, Moè, Stupnisky, & Lichtenfeld, 2019; Vierhaus, Lohaus, & Wild, 2016). This is consistent with detrimental trends that are typical of motivational constructs such as achievement goals, beliefs about control, or academic self‐concept (Bouffard, Boileau, & Vezeau, 2001). We know, therefore, that at the end of primary school students’ boredom begins to be a salient affective experience for language learning, but we do not know how it affects reading comprehension skills in the case of online reading tasks, which are typically more appealing and attractive compared with traditional reading tasks.

Primary‐school students, unlike adults, report a preference for reading on screen than on paper (Golan, Barzillai, & Katzir, 2018). This has been explained by the fact that nowadays children become familiar with digital devices at an earlier stage than did adults (Harrison & McTavish, 2018). Moreover, children’s experience of reading on paper, although less extensive than that of adults, mainly regards learning activity, while they use technology mainly in leisure time. Reading on a screen, therefore, is perceived as more attractive than reading on paper.

Boredom can be conceptualized as a functional emotion with a twofold advantage: It is informative on how current situations are unsatisfactory and it simultaneously motivates people towards desired goals, fostering regulatory processes (Elpidorou, 2017). However, as an epistemic emotion impairing both cognitive and metacognitive strategies (Muis, Chevrier, & Singh, 2018), boredom associated with reading activities may have a high impact on the integrative processes involved in online multiple‐text comprehension. This may be due to the complexity per se of the processes required to construct a coherent representation of partly conflicting texts on the same topic (Rouet et al., 2017; Stadtler & Bromme, 2013).

#### The control‐value theory

Pekrun’s CVT of achievement emotions (Pekrun, 2006) is a very useful theoretical framework to conceptualize boredom; it takes into account a variety of correlates, which play the role of antecedents or outcomes of boredom itself. In the last decades, the CVT was seminal for research on the role of emotions within the school context, given their relevance for students’ well‐being and performance (Pekrun, 2006). The CVT is an integrated motivational and information‐processing theory of the antecedents and outcomes of achievement emotions, defined as those emotions associated with learning activities or outcomes (Pekrun, 2006). They comprise at least two underlying dimensions: valence (positive and negative emotions) and activation (activating and deactivating emotions). In line with this classification, boredom can be categorized as a negative deactivating emotion. In other terms, it is an achievement emotion characterized by negative valenced feelings and low arousal, together with lack of interest and stimulation (Camacho‐Morles et al., 2021). Moreover, it results to be associated with both tasks that under‐challenge students and tasks that over‐challenge them (Krannich et al., 2019).

Among the crucial antecedents of achievement emotions, control and value appraisals play a central role. Beliefs about control refer to students’ beliefs about their control of their own actions and the corresponding outcomes, while beliefs about value refer to the degree to which students perceive that a task and/or its outcomes are important to themselves (Pekrun, 2006). According to the CVT, it is expected that low control and low value are associated with high boredom; nevertheless, also a very high control could be linked to increases in boredom. In the former case, boredom would be related to students’ perception of a task as over‐challenging, while in the latter case as under‐challenging (Acee et al., 2010). In turn, achievement emotions influence performance. Empirical data from cross‐sectional and longitudinal studies involving primary‐school to university students indicate that positive emotions are linked with better performance, while negative emotions with poorer performance (Camacho‐Morles et al., 2021; Loderer et al., 2020; Pekrun, 2006, 2017; Pekrun et al., 2017). Negative deactivating emotions (such as boredom) usually have a detrimental impact on performance, by undermining attention, motivation, and learning strategy. Moreover, the CVT postulates that achievement emotions are domain‐specific as several studies have documented for different subjects (Camacho‐Morles et al., 2021; Loderer et al., 2020; Pekrun, 2006).

Resorting to the principle of parsimony, the CVT also proposes the relative universality of the connections between achievement emotions and their correlates (Pekrun, 2006). The links between antecedents, achievement emotions, and outcomes are assumed to be the same in different individuals across genders, achievement settings, and cultures. Nevertheless, achievement emotions can differ in their rates, contents, or parameters like intensity according to such variables. For example, taking into account achievement settings, some studies revealed differences among primary‐school students, documenting that boredom was lower for evaluative settings, such as tests, compared to non‐evaluative settings, such as homework and lessons (Authors, 2013). Beyond being distinguished for their different focus on the evaluative aspect – higher for tests – test and homework settings are strictly connected. For example, for tenth‐ and twelfth‐graders, the amount of homework for subjects such as reading, mathematics, and science are significantly correlated with test scores, especially for females (Mau & Lynn, 2000). However, in primary schools usually teachers’ aims for assigning homework are not explicitly focused on increasing test performance, but mostly on developing young students’ management of time and reviewing class materials (Muhlenbruck, Cooper, Nye, & Lindsay, 1999).

In educational contexts, research on boredom is growing and it has been frequently conducted with the CVT as the main theoretical framework. However, participants are generally secondary‐school or university students. Moreover, in line with the assumptions of the CVT, most of the studies have been conducted considering different domains separately, mostly mathematics and more rarely science, language, and its sub‐domains such as reading, or in a technology‐based learning environment (Camacho‐Morles et al., 2021; Loderer et al., 2020; Tze et al., 2016).

#### Appraisals and outcomes of boredom

Concerning the relations of primary‐school students’ boredom with its control and value appraisals, some researchers have confirmed the contribution of control and value appraisals in predicting boredom at school in a variety of domains (for an overview, see Pekrun, 2017). Among the studies that investigated the relations between control and value appraisals and boredom, those about mathematics usually reported negative links, even when distinguishing the impact of different types of value, that is, intrinsic value, attainment, and utility. Some studies identified negative relations between control and boredom but not between value and boredom, or vice versa (Di Leo, Muis, Singh, & Psaradellis, 2019; Lichtenfeld, Pekrun, Stupnisky, Reiss, & Murayama, 2012; Putwain, Becker, Symes, & Pekrun, 2018; Putwain, Schmitz, Wood, & Pekrun, 2021). In the domain of reading, only negative relations between boredom and control, but not between boredom and value, emerged (Zaccoletti et al., 2020).

As regards the outcomes of boredom, the debate is still open. On the one hand, being bored can make students less attentive, effortful, and engaged, with consequent detrimental effects on performance. On the other hand, boredom can foster creative thoughts, focusing students’ attention towards more appealing tasks than the current ones (Camacho‐Morles et al., 2021). Some recent meta‐analyses have been conducted with the aim of disambiguating these findings at different educational levels. Overall, they reported a negative relation of boredom with academic performance, motivation, and learning strategies, across several domains such as mathematics, physics, and native language (Camacho‐Morles et al., 2021; Tze et al., 2016), but this relation did not emerge in technology‐based learning environments (Loderer et al., 2020).

When considering the studies with primary‐school children, findings are not always consistent. For example, in mathematics boredom is linked to academic performance as evidenced by small to moderate negative correlations (ranging from −.16 to −.42) as well as by small positive correlations (Di Leo et al., 2019; Lichtenfeld et al., 2012; Muis, Psaradellis, Lajoie, Di Leo, & Chevrier, 2015; Putwain et al., 2018; Raccanello et al., 2019). In other cases, researchers found that achievement predicted boredom but not vice versa (Putwain et al., 2021; Putwain, Wood, & Pekrun, 2020). For science learning, small negative correlations emerged (Obergriesser & Stoeger, 2016). In the domain of language and its sub‐domains, there are moderate correlations for literacy (Muis, Ranellucci, Trevors, & Duffy, 2015) but also no correlations for learning in native language or reading (Raccanello et al., 2019; Zaccoletti et al., 2020). For technology‐based learning environments, Robertson et al. (2004) reported correlations near zero between boredom and performance in the domain of writing in interactions with intelligent tutoring systems. A study by Patrick, Skinner, and Connell (1993) revealed also that boredom was associated with less motivation and effort, while Muis, Psaradellis, et al. (2015) documented negative relations between boredom and planning, deep processing strategies, and other metacognitive strategies in mathematics problem‐solving. However, overall there is scarce knowledge regarding the interplay of reading‐related boredom, control, and value appraisals and its outcomes, specifically when considering young students’ online reading comprehension, in particular of multiple texts on the same topic.

#### Word‐reading fluency

Finally, given that cognitive abilities are crucial factors in the interplay between emotions and learning (Pekrun, 2006), we examined the contribution of a basic cognitive ability, such as word‐reading fluency, on the relations between control and value appraisals, boredom, and online multiple‐text comprehension. According to the CVT, cognitive resources are one of the factors to be examined considering the relation between achievement emotions and performance. Exploring the role of word‐reading fluency is particularly salient because of the unique contribution of this basic factor, as well as that of more complex cognitive factors, which varies according to primary‐school students’ single‐text comprehension level (Language and Reading Research Consortium & Logan, 2017). Previous work showed that word‐reading fluency was a unique contributor when students were classified as having low, not high, reading comprehension skills. For skilled readers, other complex cognitive skills (e.g., comprehension monitoring) played a more critical role. As regards upper primary‐school students, previous findings indicated that word‐reading fluency is positively associated with their multiple‐text comprehension, once the role of other complex cognitive skills (e.g., comprehension monitoring) is taken into account (Florit et al., 2019). However, the interplay of word‐reading fluency in relation to other complex skills, such as reading‐related boredom, control, and value appraisals, has not been addressed yet.

Taken together, these findings solicit to investigate the possible role of a basic cognitive ability such as word‐reading fluency in moderating the relations between boredom and, respectively, its control and value antecedents and associated outcomes. Specifically, the extent to which word‐reading fluency is mastered as a basic cognitive component could modulate how boredom contributes to text comprehension. In other terms, the impact of reading‐related boredom on online multiple‐text comprehension could be more pronounced when the basic cognitive ability is already well‐mastered. However, an open issue is whether also the relation between control and value appraisals and boredom can be moderated by this basic cognitive ability, given the theoretical assumptions on the universality of the mechanisms linking appraisals and boredom in different contexts (Pekrun, 2006) and the empirical evidence supporting these negative relations. If we consider primary‐school students as reliable sources for self‐evaluating their inner states and perceptions (Nett, Daschmann, Goetz, & Stupnisky, 2016), and specifically their control about their reading skills, we could speculate that different levels of word‐reading fluency should not impact such relations.

### The current study

To sum up, there are at least four gaps that solicited our research on boredom with primary‐school students. (1) First, previous literature has not tested yet the CVT in the sub‐domain of reading in relation to technology‐based learning environments, like in the case of online multiple‐text comprehension. This ability has largely been examined in relation to various individual and contextual factors (Barzilai et al., 2018; Bråten et al., 2013; Rouet et al., 2017) and knowledge on its unexamined correlates assumes a pivotal relevance in a historical period in which most of the students worldwide have been forced to online teaching due to the COVID‐19 pandemic. (2) Second, no study investigated the correlates of boredom regarding online multiple‐text comprehension in primary‐school students. To extend knowledge on CVT and boredom in general, it is essential to include samples of younger students. This is particularly relevant not only theoretically, but also at an applied level, given that the CVT gives specific suggestions on how to improve students’ achievement through their emotions and corresponding appraisals. So, before generalizing suggestions concerning secondary‐school and university students to primary‐school children, it is necessary to test whether the underlying theoretical framework is confirmed. (3) Third, no previous study examined whether the relations of boredom with antecedents and outcomes as described earlier are moderated by basic reading abilities such as word‐reading fluency. (4) Fourth, scarce attention has been paid to studying these issues taking into account different settings for school activities, such as homework or tests.

#### Aims and research questions

The study aimed to investigate how reading‐related control and value appraisals are linked to reading‐related boredom, and how this emotion, in turn, is linked to online multiple‐text comprehension in primary‐school students. We also focused on the moderating role of word‐reading fluency on such relations. Given that achievement emotions and their appraisals are domain‐specific, we examined reading‐related boredom concerning two separate settings: one non‐evaluative, homework, and the other evaluative, tests. Two main research questions (RQ) guided the study.

*RQ1*. Does reading‐related boredom mediate the relation between reading‐related control and value appraisals and multiple‐text comprehension? Based on the aforementioned literature (Di Leo et al., 2019; Lichtenfeld et al., 2012; Muis, Ranellucci, et al., 2015; Obergriesser & Stoeger, 2016; Putwain et al., 2018, 2021; Raccanello et al., 2019; Zaccoletti et al., 2020), we hypothesized control and value appraisals to be negatively linked to boredom for homework and tests (Hypothesis 1), and boredom to be negatively linked to online multiple‐text comprehension (Hypothesis 2). Both hypotheses were monodirectional. We also examined the direct relations between antecedents and multiple‐text comprehension, underexplored in the current literature.

*RQ2*. Does students’ word‐reading fluency moderate the described relations? We explored whether high and low word‐reading fluency moderate the relations between control and value appraisals and boredom, and between boredom and multiple‐text comprehension.

## Method

### Participants

We involved a convenience sample of 334 fourth‐ (*n* = 167) and fifth‐graders (*n* = 167) from eight primary schools in Northern Italy (*M*_age_ = 10.00 years; *SD* in months = 7.54; 55% girls), located in middle‐class socio‐economic areas. The students came from 18 classes. According to their teachers’ reports, none of the children had cognitive impairments or learning disabilities, and none had been referred to the Health Services for treatment.

Written parental consent was required for participation. The study obtained the approval from the University Ethics Committee.

### Materials

Students were required to read three online texts discussing the effects of videogames and told to imagine that a friend had asked him/her some help to understand whether playing videogames is beneficial or not. The texts and coding scheme were informed by previous research in this area (Bråten, Anmarkrud, et al., 2014; Mason, Junyent, & Tornatora, 2014) and used in printed form as in previous studies (Florit, Cain, & Mason, 2019). The texts on each topic included partially conflicting information and were written by sources differing for expertise and stance: a psychologist, a parent, and a videogame creator. The first and more reliable source mainly underlined negative effects and the less reliable sources promoted mostly or exclusively positive effects. Texts appeared as clickable links in a Google search output page but they were offline. The order of appearance of the links/texts was randomized. Children read the texts in their preferred order and at their own pace since there was no time limit. They were instructed to read all the texts, to access each text as many times as they deemed necessary, and to press an ‘end task’ icon when they had completed their reading. Children received information on how to navigate across texts and completed a practice trial. During the reading of the texts, children were allowed to ask questions concerning words that were not clear.

This work is part of a larger project on the role of motivational and emotional factors, memory for source and processing, and online multiple‐text comprehension.

### Measures

#### Control and value appraisals

We assessed reading‐related control using nine items (e.g., *It is easy for me to understand the content of a text in my school reading text*) and task‐value using 11 items (e.g., *I think it is always important to understand what I read in the school reading book*) adapted from Bråten et al. (2013). Items had to be rated on a five‐point scale (1 = *not at all true for me* and 5 = *completely true for me*).

#### Boredom

We used the Achievement Emotions Questionnaire‐Elementary School (AEQ‐ES; Raccanello et al., 2019; Lichtenfeld et al., 2012) adapted for the reading domain, with items on boredom for homework (three items; e.g., *Homework in which I have to understand a text bores me to death*) and tests (four items; e.g., *Tests in which I have to understand a text bore me*). Items had to be rated on a five‐point scale (1 = *not at all* and 5 = *very much*).

#### Word‐reading fluency

We used the word‐reading task from the Italian Test Battery for the Evaluation of Developmental Dyslexia and Dysorthography (Sartori, Job, & Tressoldi, 2007). We asked students to read 112 words without errors and as fast as they could. Based on the median value of word‐reading fluency (i.e., number of syllables read per second), we distinguished students with low versus high abilities (see DeCoster, Gallucci, & Iselin, 2011, for similar approaches).

#### Online multiple‐text comprehension

After reading the three texts, children were asked to handwrite a short essay to answer the question ‘Are videogames beneficial or not?’, based on the information read in the texts. The online texts were not accessible after the reading phase but children had the possibility to ask about information explicitly presented in the texts. This approach was implemented so that children’s memory load would be reduced.

Essays were coded for the argument about the effects of videogames. The argument provided a global index of deep comprehension, reflecting whether participants considered and integrated the two different perspectives presented within texts. A five‐point scale was used: 0 = *no response/no relevant information*; 1 = *identification of a single perspective*; 2 = *identification of both perspectives*; 3 = *identification of both perspectives with explanations for one or both of them*; 4 = *identification and comparison of both perspectives with explanations for one or both and justifications for supporting one perspective*. We used an argument score as it has been shown to be positively related to more specific measures of multiple‐text comprehension such as the number of literal and inferential units in short essays and the number of texts mentioned (Authors, 2020; Barzilai & Zohar, 2012; Bråten, Ferguson, Strømsø, & Anmarkrud, 2014; Diakidoy, Mouskounti, & Ioannides, 2011). Argument score, therefore, captures children’s ability to consider, comprehend, and integrate information from texts (i.e., writing quality) rather than writing productivity.

All essays were coded by three post‐graduate students trained by the second author, and 30% of the essays on each topic were also coded by the same author independently. The overall agreement was very good (Cohen’s kappa = .90, *SE* = .05; 95% CI = [0.81–0.99]). Each disagreement was examined and discussed until consensus was reached.

### Procedure

Two researchers gathered the data over three consecutive weeks, once a week, in group sessions lasting about 1 hr, for a total of 3 hr. The students were assessed for control and task‐value in a first session, and boredom and word‐reading fluency in a second session. Each measure was preceded by a familiarization phase, including instructions and examples on how to answer. In a third session, children were administered the online multiple‐text comprehension measure in a quiet school room, which was equipped with a computer for each of them. For all measures, researchers read all the instructions and the items aloud. Children were told that there were no right or wrong answers and to answer truthfully. Socio‐demographic data were reported by parents when completing the consent forms. All the verbal labels and the pictorial material used in the instruments were appropriate to children’s gender, with male and female versions, to favour their identification.

### Data analysis

We first examined the patterns of missing data and skewness and kurtosis values. We then conducted confirmatory factor analyses (CFA) for control, task‐value, and boredom. We considered the comparative fit index (CFI), the root‐mean‐square error of approximation (RMSEA), and the standardized root mean square residual (SRMR), with CFI ≥ .90 (Marsh, Hau, & Grayson, 2005), RMSEA ≤ .08, and SRMR ≤ .10 as threshold values (Kline, 2016). We calculated Pearson bivariate correlations and descriptive statistics for all the variables (Table 1). We preliminarily checked whether boredom varied according to grade level and setting through a repeated measures analysis of variance (ANOVA), using IBM SPSS Statistics Version 27. We used Mplus Version 7 to conduct path analyses and to test the mediational role of boredom between control/task‐value and online multiple‐text comprehension, separately by setting (homework, tests). The ratio between number of observations and number of parameters (7:1) was adequate for running path analyses (Kline, 2016). In preliminary models, we included all direct and indirect paths, and then we repeated them deleting all non‐significant paths (except for the links between boredom and online multiple‐text comprehension as they involve the key outcome variable). We ran the models using *z*‐scores, given that the measures had different ranges. Finally, we performed a multi‐group analysis to test the moderation effect of word‐reading fluency.

**Table 1:** Intercorrelations, means (*M*), standard deviations (*SD*), and confidence intervals (CI) of all the examined variables

Variable	1	2	3	4	5	6
1. Reading‐related control	–					
2. Reading‐related task‐value	.37***	–				
3. Homework‐related boredom for reading	−.21***	−.32***	–			
4. Test‐related boredom for reading	−.29***	−.39***	.77***	–		
5. Word‐reading fluency	.11*	−.01	.02	.01	–	
6. Online multiple‐text comprehension	.22***	.18**	−.05	−.11*	.21***	–
*M*	3.51	4.18	2.61	2.46	3.60	1.64
*SD*	0.56	0.52	1.16	1.09	2.20	0.89
*CI*	3.45–3.57	4.12–4.23	2.48–2.73	2.35–2.58	3.36–3.84	1.54–1.73

Note.

*N* = 334.

*p < .05, **p < .01, ***p < .001.

## Results

### Preliminary analyses

The maximum percent of missing values for items was 3%. Little’s MCAR test indicated that data were completely missing at random, χ^2^(171) = 159.77, *p* = .721. The mean values of skewness and kurtosis for control, task‐value, and boredom for each item did not exceed 2.0 and 7.0 (Table 1), respectively, supporting normality assumptions (Curran, West, & Finch, 1996).

The CFA confirmed the mono‐factorial structure for control and task‐value, and the bi‐factorial structure for boredom; all factor loadings were positive and statistically significant, with good fit indexes (control: *χ^2^* (27) = 70.84, *p* < .001, CFI = .927, RMSEA = .070 (.050–.090), SRMR = .046; task‐value: *χ^2^* (43) = 76.73, CFI = .916, RMSEA = .049 (.030–.066), SRMR = .046; boredom: *χ^2^* (41) = 85.51, *p* < .001, CFI = .985, RMSEA = .057 (.040–.074), SRMR = .029).

We then performed a 2 (fourth‐graders, fifth‐graders) × 2 (homework, tests) repeated measures ANOVA on boredom, with grade level as the between‐subject factor and setting as the within‐subject factor. The effect of setting, *F*(1, 326) = 11.005, *p* = .001, *η^2^* = .03, resulted to be significant: Boredom was more intense for homework (*M* = 2.60, *SD* = 1.16) than tests (*M* = 2.46, *SD* = 1.09).

### Relation between control and value appraisals and online multiple‐text comprehension (RQ1)

**Figure 1:**
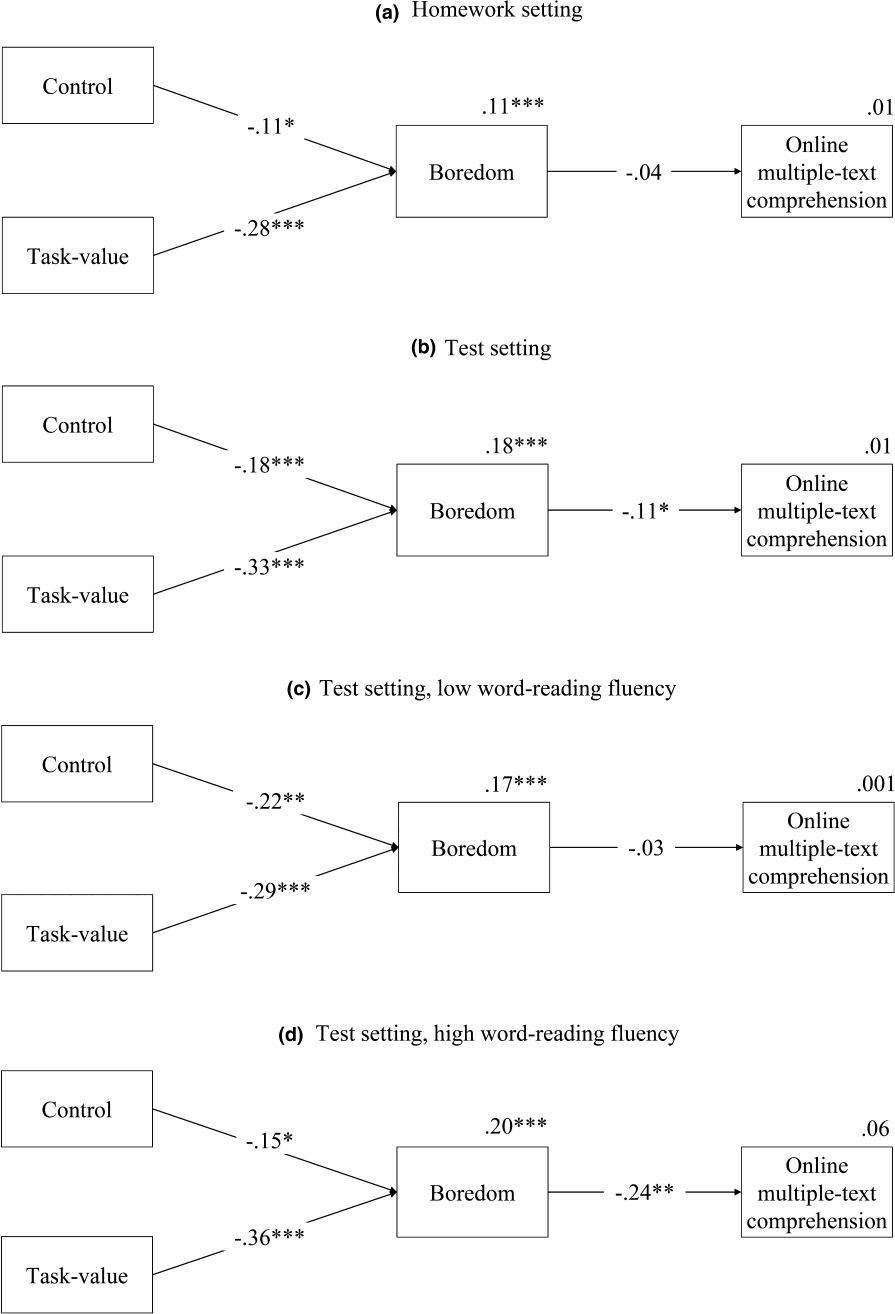
Path analyses for relations between control, task‐value, boredom, and online multiple‐text comprehension for (a) homework setting; (b) test setting; (c) tests and low word‐reading fluency; (d) tests and high word‐reading fluency. Indices for explained variance are reported next to each dependent variable. *p < .05, **p < .01, ***p < .001.

A preliminary path analysis indicated that the direct effects of control (*β* = .18, *p* = .001) and task‐value (*β* = .11, *p* = .051) on online multiple‐text comprehension were significant. Then we conducted two path analyses (Figure 1a,b) to test the indirect effects of the two appraisals on online multiple‐text comprehension through boredom, separately for setting (homework, tests). For each setting, control (homework: *β* = −.11, *p* = .047; tests: *β* = −.18, *p* = .001, respectively) and task‐value (*β* = −.28, *p* < .001; *β* = −.33, *p* < .001) were negatively associated with boredom. However, only test‐related boredom (*β* = −.11, *p* = .046) was negatively linked to online multiple‐text comprehension. The indirect effect through boredom was .02, *p* = .091, for control and .04, *p* = .057, for task‐value. The mediation was partial because when we included the direct effect of task‐value and control on online multiple‐text comprehension, we found a statistically significant path for control (*β* = .21, *p* < .001) which weakened the path from boredom to online multiple‐text comprehension.

### Moderation of word‐reading fluency in the relation between control and value appraisals, boredom, and online multiple‐text comprehension (RQ2)

To deeply explore the mediational role of boredom, we examined the moderating role of word‐reading fluency (Figure 1c,d). For low reading fluency, control (*β* = −.22, *p* = .003) and task‐value (*β* = −.29, *p* < .001) were negatively related to boredom, but the path from boredom to online multiple‐text comprehension was not significant. For high reading fluency, control (*β* = −.15, *p* = .048) and task‐value (*β* = −.36, *p* < .001) were negatively linked to boredom, and this emotion negatively predicted online multiple‐text comprehension (*β* = −.24, *p* = .002). When we included the direct effect of task‐value and control, nothing changed in the model for low reading fluency, but for high reading fluency again, only control directly predicted online multiple‐text comprehension (*β* = .16, *p* = .046). The inclusion of this path weakened again the link between boredom and online multiple‐text comprehension (*β* = −.14, *p* = .088). In other words, boredom partially mediated the relation between control and online multiple‐text comprehension (indirect effect: .04, *p* = .095), and totally the relation between task‐value and the outcome variable (indirect effect: .09, *p* = .009).

## Discussion

Our first research question aimed at exploring the mediating role of boredom in the relation between control and value appraisals and online multiple‐text comprehension (RQ1). The results confirm the key role of control and value as antecedents of boredom for reading in primary school, in line with the theoretical expectations of the CVT (Pekrun, 2006) and supporting Hypothesis 1. Studies with primary‐school students have corroborated the assumptions of the CVT on the relation between control and value appraisals and boredom in the domain of mathematics (Di Leo et al., 2019; Lichtenfeld et al., 2012; Putwain et al., 2018, 2021). In contrast, previous findings on reading have documented negative relations with boredom only for control (Zaccoletti et al., 2020). However, in that case, the comprehension of single printed texts was considered and participants were young adolescents in lower secondary school. In addition, the authors did not distinguish different settings while our analyses considered separately the two settings of homework and tests. Therefore, our data suggest that if we examine specific settings separately – in line with the CVT’s assumptions on the domain‐specificity of achievement emotions and their antecedents (Pekrun, 2006) – the phenomena pertaining to the complex interrelations between boredom and its correlates can be caught with a higher level of granularity.

Moreover, our analyses indicate that boredom had disruptive effects on online multiple‐text comprehension only in the evaluative setting, that is, testing, partially confirming Hypothesis 2. These findings contribute to shed light on how boredom impacts performance operationalized in terms of online multiple‐text comprehension, an ability that has assumed a paramount relevance also in light of the unexpectedly recent changes in education due to the current pandemic. The detrimental effects of boredom on performance can be related to the influence of its antecedents. For the test setting, our results suggest that a low control about reading abilities combined with a low task‐value results in higher boredom, which in turn can be associated with reduced engagement and lower investment of attentional resources on reading tasks (Muis, Psaradellis, et al., 2015). We can also speculate that the impact of boredom is not related to the particular salience of this emotion in the examined context; indeed, boredom was more intense for the non‐evaluative setting of homework compared with the evaluative setting of tests, consistently with our expectations. This is in line again with the theoretical assumptions of the CVT (Pekrun, 2006), according to which the relations between achievement emotions and their correlates are universal, but their rates can vary according to a variety of other aspects, such as the context (operationalized, in our case, in terms of settings).

Moreover, it is worth noting that detecting different links between boredom and performance in the two examined settings can also shed some light on the reasons underlying some inconsistent results of previous research involving primary‐school students, in which boredom was not examined according to different settings (Di Leo et al., 2019; Lichtenfeld et al., 2012; Muis, Psaradellis, et al., 2015; Muis, Ranellucci, et al., 2015; Obergriesser & Stoeger, 2016; Putwain et al., 2018, 2020, 2021; Raccanello et al., 2019; Robertson et al., 2004; Zaccoletti et al., 2020). Distinguishing homework and tests might have helped to identify another moderator of the relations between boredom and performance, that is, setting, which has been frequently neglected in previous literature (for an exception, see Raccanello et al., 2013). The impact of boredom on online multiple‐text comprehension is less marked for the homework setting because in that case students are frequently helped by adults and can ask for their support. This diminishes the prominence of the need to protect their self and the perception of their efficacy in the homework task. Moreover, a role might be played by the challenges associated with a task, given that boredom increases in case of both under‐ and over‐challenging tasks, and in turns influences outcomes such as career aspirations (Acee et al., 2010; Krannich et al., 2019). However, only for over‐challenged students (and not under‐challenged) there is a negative impact on academic self‐concept, at least for secondary‐school students (Acee et al., 2010; Krannich et al., 2019). Future studies should examine further the moderating role of task challenges in the relations between boredom, control and value appraisals, and performance in young students.

Another explanation concerning the links between boredom and decreased multiple‐text comprehension could consider the reciprocal relations between achievement emotions and performance as assumed by Pekrun’s model (Pekrun, 2006), taking also into account readers’ tendency to process digital information superficially (Clinton, 2019; Delgado et al., 2018). Indeed, previous research has indicated that also in primary‐school children boredom is frequently associated with less motivation and effort, poorer planning abilities, and a lower use of deep and/or other metacognitive strategies (Muis, Psaradellis, et al., 2015; Patrick et al., 1993). All this could play a role in processing information in a careless way, thus influencing performance. According to the shallow hypothesis (Annisette & Lafreniere, 2017), today’s students are more inclined towards superficial processing when reading digitally than in print, which accounts for the decreased deep comprehension in digital environments compared to traditional reading medium (Clinton, 2019; Delgado et al., 2018). Therefore, boredom could also be due to the awareness of high difficulty of a reading task in front of unfulfilled expectations of low difficulty.

Our second research question regarded the possible moderation effect of students’ word‐reading fluency in the relations between antecedents, boredom, and multiple‐text comprehension (RQ2). The results shed light on word‐reading fluency as a cognitive mechanism through which boredom influences performance. For both settings, control and value appraisals were again negatively related to boredom. However, online multiple‐text comprehension is affected by test‐related boredom only when basic word‐reading abilities are well‐mastered. In other terms, our findings indicate that word‐reading abilities are not only precursors of more complex cognitive abilities concerning the comprehension of printed texts (Florit et al., 2019; Language and Reading Research Consortium & Logan, 2017), but they also contribute to the intricate patterns of interrelations between cognition and affect in the comprehension of online multiple texts. In line with previous literature, affective factors should be taken into account to explain variance in complex comprehension tasks beyond basic cognitive abilities such as word‐reading fluency, at higher levels of reading ability, but not at lower levels. Affective factors influence children’s criteria (i.e., standards of coherence; van den Broek et al., 2011) for establishing how coherent their understanding of a passage should be, particularly in a testing situation. Our findings, therefore, suggest that as basic word‐reading skills are mastered, variability in children’s test‐related boredom comes into play to set different standards of coherence in reading activity, and, as a result, determines variability in children’s comprehension. To this regard, this study contributes to more explicitly specifying how individual difference factors affect the preparatory or ‘default stance’ towards multiple‐text comprehension (List & Alexander, 2019). In brief, the infusion of affect on cognitive performance is moderated by specific cognitive abilities, as assumed by the CVT (Pekrun, 2006).

### Educational implications

The findings about the relations between control and value appraisals, boredom, and online multiple‐test comprehension have practical implications for fostering students’ performance, as comprehension of complex information presented online is required at school. The CVT offers a viable way to improve students’ performance – in our case online multiple‐text comprehension – through a variety of channels. Traditionally, the main aim of education is to promote learning focusing on the improvement of cognitive and metacognitive abilities. However, when practices aiming at directly impacting students’ performance fail or are not sufficient to produce the desired changes, knowledge of the influence of achievement emotions on performance, and knowledge about the impact of control and value appraisals on achievement emotions, become a priority. Specifically, teachers interested in supporting students’ online multiple‐text comprehension can work both to reduce reading‐related boredom and to improve control and value beliefs. On the one hand, to reduce boredom, they can act, for example, as models actively interested in the tasks students must deal with. They can increase their engagement in such tasks proposing particularly attractive materials (and nowadays technology offers a very wide range of possibilities) as well as anchoring task content to students’ personal interests and autobiographical experiences. On the other hand, teachers can work directly to reframe negative beliefs that are antecedents of boredom (e.g., Dresel & Haugwitz, 2008; for retraining attributional interventions, see Perry, Stupnisky, Hall, Chipperfield, & Weiner, 2010), in particular improving students’ beliefs on their control and value about reading activities.

In addition, the data on the detrimental nature of boredom in evaluative tests drives teachers’ attention towards controlling task characteristics that can be associated with boredom, such as low or high level of easiness, and novelty of contents, when devising assessment practices. Moreover, previous findings described a gradually decreasing emotional support from teachers across the school years (Vierhaus et al., 2016). Therefore, this solicits actions aimed to favour more steady levels of teachers’ support in the classroom. This may be implemented, for example, involving teachers in a training on the awareness of the role played by achievement emotions and related regulation abilities within learning contexts, including antecedents of achievement emotions. Teachers could be directly involved in the conduction of interventions on these issues with the students (e.g., Raccanello & Hall, 2020). An adequate support in turn may prevent from increasing reading‐related boredom, with positive outcomes also for cognitive performance.

The results on the role of word‐reading fluency as moderator of the investigated relations also have relevant practical implications. Teachers and other learning‐focused professionals need to be aware of these effects when planning interventions to sustain students’ emotional and cognitive antecedents of a good performance. As students become more competent and master basic cognitive abilities such as word‐reading fluency, teachers can gradually diminish the level of their scaffolding in reading tasks. However, the key role of teachers as guides for students’ learning continues to be relevant, although modified to smoothly adapt it to the changes in students’ competencies. In other words, our findings suggest to flexibly adapt the level of scaffolding offered to students in relation to a given task. Specifically, when students master word‐reading fluency, teachers should pay particular attention to emotional aspects such as those related to boredom. Moreover, our findings represent a starting point to explore whether the assumptions of the CVT are also confirmed when cognitive abilities are impaired, for example in children with reading difficulties.

### Limitations

This study suffers from limitations related to the self‐report nature of the data, such as desirability biases or memory distortions, and the reduced sample size. Nevertheless, self‐report instruments are still among the privileged ways to have access to individuals’ inner states. Future studies can generalize our findings by involving larger samples and using experience sample methods for assessing the dynamics of reading‐related boredom. In addition, the role of boredom together with the role of other achievement emotions can be examined taking into account a larger variety of technological contexts and tasks, for example when students use educational web applications. Moreover, we did not test a model in which multiple‐text comprehension predicted boredom; future studies could address this issue. Finally, the CVT postulates intra‐individual relations. However, due to the study’s cross‐sectional design, we could examine only inter‐individual relations. Longitudinal designs are needed for a more complete investigation.

### Conclusions

Notwithstanding these limitations, our study contributes to extend current knowledge on the role of cognitive and affective factors, and particularly of a neglected emotion such as boredom, on online multiple‐text comprehension. Such factors are crucial for knowledge acquisition in both school‐related and everyday activities, characterizing the challenges posed to students in our current technological society. From a theoretical perspective, our findings support the central role of reading‐related boredom with respect to an ability that is increasingly relevant in todays’ information society, that is, the comprehension of multiple texts on the same topic. Considering the CVT as the main theoretical framework (Pekrun, 2006), we demonstrate the mediating role of reading‐related boredom in the relations between reading‐related control and value appraisals and online multiple‐text comprehension, as well as the moderating role of word‐reading fluency in such relations. This last issue, neglected by previous research, is particularly relevant to understand the complex pattern of interactions between various factors in learning contexts. The issue enables to better appreciate how similar emotions felt by students with different cognitive abilities can impact their performance differently not only in reading but also in other domains of school learning. Such knowledge acquires an increasing relevance in the current educational context in which the use of technology for learning has markedly increased.

## Conflicts of interest

All authors declare no conflict of interest.

## Data Availability

The data that support the findings of this study are available from the corresponding author upon reasonable request.

## References

[bjep12448-bib-0001] Acee, T. W. , Kim, H. , Kim, H. J. , Kim, J.‐I. , Chu, H.‐N.‐R. , Kim, M. , … Wicker, F. W. (2010). Academic boredom in under‐ and over‐challenging situations. Contemporary Educational Psychology, 35(1), 17–27. 10.1016/j.cedpsych.2009.08.002

[bjep12448-bib-0002] Annisette, L. E. , & Lafreniere, K. D. (2017). Social media, texting, and personality: A test of the shallowing hypothesis. Personality and Individual Differences, 115, 154–158. 10.1016/j.paid.2016.02.043

[bjep12448-bib-0003] Arfé, B. , Dockrell, J. E. , & De Bernardi, B. (2016). The effect of language specific factors on early written composition: The role of spelling, oral language and text generation skills in a shallow orthography. Reading and Writing: an Interdisciplinary Journal, 29, 501–527. 10.1007/s11145-015-9617-5

[bjep12448-bib-0004] Baker, R. S. J. , Walonoski, J. , Heffernan, N. T. , Roll, I. , Corbett, A. , & Koedinger, K. R. (2008). Why students engage in “gaming the system” behavior in interactive learning environments. Journal of Interactive Learning Research, 19, 185–224.

[bjep12448-bib-0005] Barzilai, S. , & Zohar, A. (2012). Epistemic thinking in action: Evaluating and integrating online sources. Cognition and Instruction, 30(1), 39–85. 10.1080/07370008.2011.636495

[bjep12448-bib-0006] Barzilai, S. , Zohar, A. R. , & Mor‐Hagani, S. (2018). Promoting integration of multiple texts: A review of instructional approaches and practices. Educational Psychology Review, 30, 973–999. 10.1007/s10648-018-9436-8

[bjep12448-bib-0007] Bouffard, T. , Boileau, L. , & Vezeau, C. (2001). Students’ transition from elementary to high school and changes of the relationship between motivation and academic performance. European Journal of Psychology of Education, 16, 589–604. 10.1007/BF03173199

[bjep12448-bib-0008] Bråten, I. , Anmarkrud, Ø. , Brandmo, C. , & Strømsø, H. I. (2014). Developing and testing a model of direct and indirect relationships between individual differences, processing, and multiple‐text comprehension. Learning and Instruction, 30, 9–24. 10.1016/j.learninstruc.2013.11.002

[bjep12448-bib-0009] Bråten, I. , Ferguson, L. E. , Anmarkrud, Ø. , & Strømsø, H. I. (2013). Prediction of learning and comprehension when adolescents read multiple texts: The roles of word‐level processing, strategic approach, and reading motivation. Reading and Writing, 26, 321–348. 10.1007/s11145-012-9371-x

[bjep12448-bib-0010] Bråten, I. , Ferguson, L. E. , Strømsø, H. I. , & Anmarkrud, Ø. (2014). Students working with multiple conflicting documents on a scientific issue: Relations between epistemic cognition while reading and sourcing and argumentation in essays. British Journal of Educational Psychology, 84(1), 58–85. 10.1111/bjep.12005 24547754

[bjep12448-bib-0011] Camacho‐Morles, J. , Slemp, G. R. , Oades, L. G. , Pekrun, R. , & Morrish, L. (2019). Relative incidence and origins of achievement emotions in computer‐based collaborative problem‐solving: A control‐value approach. Computers in Human Behavior, 98, 41–49. 10.1016/j.chb.2019.03.035

[bjep12448-bib-0012] Camacho‐Morles, J. , Slemp, G. R. , Pekrun, R. , Loderer, K. , Hou, H. , & Oades, L. G. (2021). Activity achievement emotions and academic performance: A meta‐analysis. Educational Psychology Review. Advance online publication 10.1007/s10648-020-09585-3

[bjep12448-bib-0013] Clinton, V. (2019). Reading from paper compared to screens: A systematic review and meta‐analysis. Journal of Research in Reading, 42, 288–325. 10.1111/1467-9817.12269

[bjep12448-bib-0014] Curran, P. J. , West, S. G. , & Finch, J. F. (1996). The robustness of test statistics to nonnormality and specification error in confirmatory factor analysis. Psychological Methods, 1(1), 16–29. 10.1037/1082-989X.1.1.16

[bjep12448-bib-0015] Davis, D. S. , Huang, B. , & Yi, T. (2017). Making sense of science texts: A mixed‐methods examination of predictors and processes of multiple‐text comprehension. Reading Research Quarterly, 52, 227–252. 10.1002/rrq.162

[bjep12448-bib-0016] DeCoster, J. , Gallucci, M. , & Iselin, A. M. R. (2011). Best practices for using median splits, artificial categorization, and their continuous alternatives. Journal of Experimental Psychopathology, 2, 197–209. 10.5127/jep.008310

[bjep12448-bib-0017] Delgado, P. , Vargas, C. , Ackerman, R. , & Salmerón, L. (2018). Don’t throw away your printed books: A meta‐analysis on the effects of reading media on reading comprehension. Educational Research Review, 25, 23–38. 10.1016/j.edurev.2018.09.003

[bjep12448-bib-0018] Di Leo, I. , Muis, K. R. , Singh, C. A. , & Psaradellis, C. (2019). Curiosity… Confusion? Frustration! The role and sequencing of emotions during mathematics problem solving. Contemporary Educational Psychology, 58, 121–137. 10.1016/j.cedpsych.2019.03.001

[bjep12448-bib-0019] Diakidoy, I.‐A.‐N. , Mouskounti, T. , & Ioannides, C. (2011). Comprehension and learning from refutation and expository texts. Reading Research Quarterly, 46(1), 22–38. 10.1598/RRQ.46.1.2

[bjep12448-bib-0020] Dresel, M. , & Haugwitz, M. (2008). A computer‐based training approach to foster motivation and self‐regulated learning. Journal of Experimental Education, 77(1), 3–18. 10.3200/JEXE.77.1.3-20

[bjep12448-bib-0021] Elpidorou, A. (2017). The bored mind is a guiding mind: Toward a regulatory theory of boredom. Phenomenology and the Cognitive Sciences, 17, 455–484. 10.1007/s11097-017-9515-1

[bjep12448-bib-0022] Florit, E. , Cain, K. , & Mason, L. (2019). Going beyond children’s single‐text comprehension: The role of fundamental and higher–level skills in 4th graders’ multiple‐document comprehension. British Journal of Educational Psychology, 90, 449–472. 10.1111/bjep.12288 31070262

[bjep12448-bib-0076] Florit, E. , Cain, K. , & Mason, L. (2019). Going beyond children’s single‐text comprehension: The role of word reading, working memory, comprehension monitoring and source use in 4th graders’ multiple‐document comprehension. British Journal of Educational Psychology. http://dx.doi.org/10.1111/bjep.12288

[bjep12448-bib-0023] Florit, E. , De Carli, P. , Giunti, G. , & Mason, L. (2020). Advanced theory of mind uniquely contributes to children’s multiple‐text comprehension. Journal of Experimental Child Psychology, 189, e104708. 10.1016/j.jecp.2019.104708 31634737

[bjep12448-bib-0024] Golan, D. D. , Barzillai, M. , & Katzir, T. (2018). The effect of presentation mode on children’s reading preferences, performance, and self‐evaluations. Computers & Education, 126, 346–358. 10.1016/j.compedu.2018.08.001

[bjep12448-bib-0079] Harrison, E. , & McTavish, M. (2018). ‘i’Babies: Infants' and toddlers' emergent language and literacy in a digital culture of iDevices. Journal of Early Childhood Literacy, 18(2), 163–188. 10.1177/1468798416653175

[bjep12448-bib-0025] Kim, Y. G. , & Schatschneider, C. (2017). Expanding the developmental models of writing: A direct and indirect effects model of developmental writing (DIEW). Journal of Educational Psychology, 109(1), 35–50. 10.1037/edu0000129 28260812 PMC5330285

[bjep12448-bib-0026] Kingsley, T. L. , Cassady, J. C. , & Tancock, S. M. (2015). Successfully promoting 21st century online research skills: Interventions in 5th‐grade classrooms. Reading Horizons, 54, 92–134.

[bjep12448-bib-0027] Kintsch, W. (1988). The role of knowledge in discourse comprehension: A construction‐integration model. Psychological Review, 95, 163–182. 10.1037/0033-295X.95.2.163 3375398

[bjep12448-bib-0028] Kline, R. B. (2016). Principles and practice of structural equation modeling (4th ed.). New York: The Guilford Press.

[bjep12448-bib-0029] Kong, Y. , Seo, Y. S. , & Zhai, L. (2018). Comparison of reading performance on screen and on paper: A meta‐analysis. Computers & Education, 123, 138–149. 10.1016/j.compedu.2018.05.005

[bjep12448-bib-0030] Krannich, M. , Goetz, T. , Lipnevich, A. A. , Bieg, M. , Roos, A.‐L. , Becker, E. S. , & Morger, V. (2019). Being over‐ or underchallenged in class: Effects on students’ career aspirations via academic self‐concept and boredom. Learning and Individual Differences, 69, 206–218. 10.1016/j.lindif.2018.10.004

[bjep12448-bib-0031] Lajoie, S. P. , Pekrun, R. , Azevedo, R. , & Leighton, J. P. (2020). Understanding and measuring emotions in technology‐rich learning environments. Learning and Instruction, 70, 101272. 10.1016/j.learninstruc.2019.101272

[bjep12448-bib-0032] Language and Reading Research Consortium, & Logan, J. (2017). Pressure points in reading comprehension: A quantile multiple regression analysis. Journal of Educational Psychology, 109, 451–464. 10.1037/edu0000150

[bjep12448-bib-0033] LaRusso, M. , Kim, H. Y. , Selman, R. , Uccelli, P. , Dawson, T. , Jones, S. , … Snow, C. (2016). Contributions of academic language, perspective taking, and complex reasoning to deep reading comprehension. Journal of Research on Educational Effectiveness, 9, 201–222. 10.1080/19345747.2015.1116035

[bjep12448-bib-0034] Lichtenfeld, S. , Pekrun, R. , Stupnisky, R. H. , Reiss, K. , & Murayama, K. (2012). Measuring students’ emotions in the early years: The Achievement Emotions Questionnaire‐Elementary School (AEQ‐ES). Learning and Individual Differences, 22, 190–201. 10.1016/j.lindif.2011.04.009

[bjep12448-bib-0035] List, A. , & Alexander, P. A. (2019). Toward an integrated framework of multiple text use. Educational Psychologist, 54(1), 20–39. 10.1080/00461520.2018.1505514

[bjep12448-bib-0036] Loderer, K. , Pekrun, R. , & Lester, J. C. (2020). Beyond cold technology: A systematic review and meta‐analysis on emotions in technology‐based learning environments. Learning and Instruction, 70, 101162. 10.1016/j.learninstruc.2018.08.002

[bjep12448-bib-0037] Marsh, H. W. , Hau, K.‐T. , & Grayson, D. (2005). Goodness of fit evaluation in structural equation modeling. In A. Maydeu‐Olivares & J. McArdle (Eds.), Contemporary psychometrics (pp. 275–340). Hillsdale, NJ: Erlbaum.

[bjep12448-bib-0038] Mau, W. C. , & Lynn, R. (2000). Gender differences in homework and test scores in mathematics, reading and science at tenth and twelfth grade. Psychology, Evolution & Gender, 2, 119–125. 10.1080/14616660050200904

[bjep12448-bib-0078] Mason, L. , Junyent, A. A. , & Tornatora, M. C. (2014). Epistemic evaluation and comprehension of web‐source information on controversial science‐related topics: Effects of a short‐term instructional intervention. Computers & Education, 76(1), 143–157. 10.1016/j.compedu.2014.03.016

[bjep12448-bib-0077] Mason, L. , Scrimin, S. , Zaccoletti, S. , Tornatora, M. C. , & Goetz, T. (2018). Webpage reading: Psychophysiological correlates of emotional arousal and regulation predict multiple‐text comprehension. Computers in Human Behavior, 87, 317–326. 10.1016/j.chb.2018.05.020

[bjep12448-bib-0039] Muhlenbruck, L. , Cooper, H. , Nye, B. , & Lindsay, J. J. (1999). Homework and achievement: Explaining the different strengths of relation at the elementary and secondary school levels. Social Psychology of Education, 3, 295–317. 10.1023/A:1009680513901

[bjep12448-bib-0040] Muis, K. R. , Chevrier, M. , & Singh, C. A. (2018). The role of epistemic emotions in personal epistemology and self‐regulated learning. Educational Psychologist, 53, 165–184. 10.1080/00461520.2017.1421465

[bjep12448-bib-0041] Muis, K. R. , Pekrun, R. , Sinatra, G. M. , Azevedo, R. , Trevors, G. J. , Meier, E. , & Heddy, B. C. (2015). The curious case of climate change: Testing a theoretical model of epistemic beliefs, epistemic emotions, and complex learning. Learning and Instruction, 39, 168–183. 10.1016/j.learninstruc.2015.06.003

[bjep12448-bib-0042] Muis, K. R. , Psaradellis, C. , Lajoie, S. P. , Di Leo, I. , & Chevrier, M. (2015). The role of epistemic emotions in mathematics problem solving. Contemporary Educational Psychology, 42, 172–185. 10.1016/j.cedpsych.2015.06.003

[bjep12448-bib-0043] Muis, K. R. , Ranellucci, J. , Trevors, G. , & Duffy, M. C. (2015). The effects of technology‐mediated immediate feedback on kindergarten students’ attitudes, emotions, engagement and learning outcomes during literacy skills development. Learning and Instruction, 38, 1–13. 10.1016/j.learninstruc.2015.02.001

[bjep12448-bib-0044] Nett, U. E. , Daschmann, E. C. , Goetz, T. , & Stupnisky, R. H. (2016). How accurately can parents judge their children’s boredom in school? Frontiers in Psychology, 7, 770. 10.3389/fpsyg.2016.00770 27445876 PMC4927813

[bjep12448-bib-0045] Obergriesser, S. , & Stoeger, H. (2016). The influence of emotions and learning preferences on learning strategy use before transition into high‐achiever track secondary school. High Ability Studies, 27(1), 5–38. 10.1080/13598139.2015.1100980

[bjep12448-bib-0046] Patrick, B. C. , Skinner, E. A. , & Connell, J. P. (1993). What motivates children’s behavior and emotion? Joint effects of perceived control and autonomy in the academic domain. Journal of Personality and Social Psychology, 65, 781–791. 10.1037/0022-3514.65.4.781 8229650

[bjep12448-bib-0047] Paul, J. , Stadtler, M. , & Bromme, R. (2019). Effects of a sourcing prompt and conflicts in reading materials on elementary students’ use of source information. Discourse Processes, 56, 155–169. 10.1080/0163853X.2017.1402165

[bjep12448-bib-0048] Pekrun, R. (2006). The control‐value theory of achievement emotions: Assumptions, corollaries, and implications for educational research and practice. Educational Psychology Review, 18, 315–341. 10.1007/s10648-006-9029-9

[bjep12448-bib-0049] Pekrun, R. (2017). Emotion and achievement during adolescence. Child Development Perspectives, 11, 215–221. 10.1111/cdep.12237

[bjep12448-bib-0051] Pekrun, R. , Lichtenfeld, S. , Marsh, H. W. , Murayama, K. , & Goetz, T. (2017). Achievement emotions and academic performance: Longitudinal models of reciprocal effects. Child Development, 88, 1653–1670. 10.1111/cdev.12704 28176309

[bjep12448-bib-0052] Perry, R. P. , Stupnisky, R. H. , Hall, N. C. , Chipperfield, J. G. , & Weiner, B. (2010). Bad starts and better finishes: Attributional retraining and initial performance in competitive achievement settings. Journal of Social and Clinical Psychology, 29, 668–700. 10.1521/jscp.2010.29.6.668

[bjep12448-bib-0053] Potocki, A. , de Pereyra, G. , Ros, C. , Macedo‐Rouet, M. , Stadtler, M. , Salmerón, L. , & Rouet, J.‐F. (2020). The development of source evaluation skills during adolescence: Exploring different levels of source processing and their relationships. Infancia Y Aprendizaje, 43(1), 19–59. 10.1080/02103702.2019.1690848

[bjep12448-bib-0054] Putwain, D. W. , Becker, S. , Symes, W. , & Pekrun, R. (2018). Reciprocal relations between students’ academic enjoyment, boredom, and achievement over time. Learning and Instruction, 54, 73–81. 10.1016/j.learninstruc.2017.08.004

[bjep12448-bib-0056] Putwain, D. W. , Wood, P. , & Pekrun, R. (2020). Achievement emotions and academic achievement: Reciprocal relations and the moderating influence of academic buoyancy. Journal of Educational Psychology. Advance online publication 10.1037/edu0000637

[bjep12448-bib-0055] Putwain, D. W. , Schmitz, E. A. , Wood, P. , & Pekrun, R. (2021). The role of achievement emotions in primary school mathematics: Control‐value antecedents and achievement outcomes. British Journal of Educational Psychology, 91(1), 347–367. 10.1111/bjep.12367 32662521

[bjep12448-bib-0057] Raccanello, D. , Brondino, M. , & De Bernardi, B. (2013). Achievement emotions in elementary, middle, and high school: How do students feel about specific contexts in terms of settings and subject‐domains? Scandinavian Journal of Psychology, 54, 477–484. 10.1111/sjop.12079 24111747

[bjep12448-bib-0058] Raccanello, D. , Brondino, M. , Moè, A. , Stupnisky, R. , & Lichtenfeld, S. (2019). Enjoyment, boredom, anxiety in elementary schools in two domains: Relations with achievement. The Journal of Experimental Education, 87, 449–469. 10.1080/00220973.2018.1448747

[bjep12448-bib-0059] Raccanello, D. , & Hall, R. (2020). An intervention promoting understanding of achievement emotions with middle school students. European Journal of Psychology of Education, 1–22. Advance online publication 10.1007/s10212-020-00498-x PMC7530548 40477149

[bjep12448-bib-0060] Robertson, J. , Cross, B. , Macleod, H. , & Wiemer‐Hastings, P. (2004). Children’s interactions with animated agents in an intelligent tutoring system. International Journal of Artificial Intelligence in Education, 14, 335–357.

[bjep12448-bib-0061] Rodrigo, M. M. T. , Baker, R. S. J. D. , Agapito, J. , Nabos, J. , Repalam, M. C. , Reyes, S. S. , & San Pedro, M. O. C. Z. (2012). The effects of an interactive software agent on student affective dynamics while using an intelligent tutoring system. IEEE Transactions on Affective Computing, 3, 224–236. 10.1109/T-AFFC.2011.41

[bjep12448-bib-0062] Rouet, J.‐F. , Britt, M. A. , & Durik, A. M. (2017). RESOLV: Readers’ representation of reading contexts and tasks. Educational Psychologist, 52, 200–215. 10.1080/00461520.2017.1329015

[bjep12448-bib-0063] Sabourin, J. L. , & Lester, J. C. (2014). Affect and engagement in game‐based learning environments. IEEE Transactions on Affective Computing, 5(1), 45–56. 10.1109/T-AFFC.2013.27

[bjep12448-bib-0064] Sartori, G. , Job, R. , & Tressoldi, P. E. (2007). Batteria per la valutazione della dislessia e disortografia evolutiva [Test battery for the assessment of developmental dyslexia and dysorthographia]. Organizzazioni Speciali.

[bjep12448-bib-0065] Schwartze, M. M. , Frenzel, A. C. , Goetz, T. , Marx, A. K. G. , Reck, C. , Pekrun, R. , & Fiedler, D. (2020). Excessive boredom among adolescents: A comparison between low and high achievers. PLoS One, 15, e0241671. 10.1371/journal.pone.0241671 33152022 PMC7644046

[bjep12448-bib-0066] Schwartze, M. M. , Frenzel, A. C. , Goetz, T. , Pekrun, R. , Reck, C. , Marx, A. K. G. , & Fiedler, D. (2021). Boredom makes me sick: Adolescents’ boredom trajectories and their health‐related quality of life. 10.31234/osf.io/zm5es PMC8296113 34200811

[bjep12448-bib-0067] Scrimin, S. , & Mason, L. (2015). Does mood influence text processing and comprehension? Evidence from an eye‐movement study. British Journal of Educational Psychology, 85, 387–406. 10.1111/bjep.12080 26010020

[bjep12448-bib-0068] Stadtler, M. , & Bromme, R. (2013). Multiple document comprehension: An approach to public understanding of science. Cognition and Instruction, 31, 122–129. 10.1080/07370008.2013.771106

[bjep12448-bib-0069] Trevors, G. J. , Muis, K. R. , Pekrun, R. , Sinatra, G. M. , & Muijselaar, M. M. L. (2017). Exploring the relations between epistemic beliefs, emotions, and learning from texts. Contemporary Educational Psychology, 48, 116–132. 10.1016/j.cedpsych.2016.10.001

[bjep12448-bib-0070] Tulis, M. , & Ainley, M. (2011). Interest, enjoyment and pride after failure experiences? Predictors of students’ state‐emotions after success and failure during learning in mathematics. Educational Psychology, 31, 779–807. 10.1080/01443410.2011.608524

[bjep12448-bib-0071] Tze, V. M. , Daniels, L. M. , & Klassen, R. M. (2016). Evaluating the relationship between boredom and academic outcomes: A meta‐analysis. Educational Psychology Review, 28(1), 119–144. 10.1007/s10648-015-9301-y

[bjep12448-bib-0072] UNESCO . (2021). Education: From disruption to recovery. Retrieved April 26, 2021, from https://en.unesco.org/covid19/educationresponse

[bjep12448-bib-0073] van den Broek, P. W. , Bohn‐Gettler, C. , Kendeou, P. , Carlson, S. , & White, M. J. (2011). When a reader meets a text: The role of standards of coherence in reading comprehension. In M. T. McCrudden , J. P. Magliano & G. Schraw (Eds.), Text relevance and learning from text (pp. 123–140). Information Age Publishing.

[bjep12448-bib-0074] Vierhaus, M. , Lohaus, A. , & Wild, E. (2016). The development of achievement emotions and coping/emotion regulation from primary to secondary school. Learning and Instruction, 42, 12–21. 10.1016/j.learninstruc.2015.11.002

[bjep12448-bib-0075] Zaccoletti, S. , Altoè, G. , & Mason, L. (2020). The interplay of reading‐related emotions and updating in reading comprehension performance. British Journal of Educational Psychology, 90, 663–682. 10.1111/bjep.12324 31654408

